# Time-saving potential of daily online adaptive proton therapy for head and neck cancers by reducing number of beams^☆^

**DOI:** 10.1016/j.phro.2025.100853

**Published:** 2025-10-15

**Authors:** Evangelia Choulilitsa, Katarzyna Czerska, Barbara Bachtiary, Damien Charles Weber, Antony John Lomax, Francesca Albertini

**Affiliations:** a Center for Proton Therapy, Paul Scherrer Institute, Villigen, Switzerland; b Department of Physics, ETH Zurich, Zurich, Switzerland,; c Department of Radiation Oncology, Inselspital, Bern University Hospital, University of Bern, Bern, Switzerland

**Keywords:** Online adaptive proton therapy, Reduced beam configuration, Fast delivery, Head and neck cancer

## Abstract

**Background and purpose:**

Standard care for head and neck cancer (HNC) treatment with proton therapy typically involves a 4–6 field Intensity Modulated Proton Therapy plan to enhance robustness towards anatomical changes and patient misalignments. This study aimed to evaluate whether a more efficient plan with fewer beams, designed for faster delivery, can be combined with online daily adaptation (DAPT) to provide treatment of comparable quality, and improve treatment outcomes.

**Materials and methods:**

We retrospectively analyzed five HNC patients with available daily 3D imaging treated at our institution. To simulate DAPT, synthetic Computed Tomography (CT) images were generated by deforming planning CT to each daily Cone-Beam CT with targets and organs-at-risk (OARs) propagated to daily images. Three plans were created per-patient: Offline_SBC_ and DAPT_SBC_ with standard, and DAPT_RBC_ with reduced, beam configuration. DAPT_SBC_ and DAPT_RBC_ were reoptimized on daily synCTs, while Offline_SBC_ followed clinical workflow, with offline replanning as needed.

**Results:**

Offline_SBC_ showed >5% target underdosage in 15% of fractions, with both adaptive approaches significantly improving coverage. Although DAPT_RBC_ outperformed Offline_SBC_ for target coverage, its advantage in OARs sparing was less definitive. DAPT_SBC_ reduced pooled average normal tissue dose across patients and fractions by 13% and pooled average normal tissue complication probability for xerostomia by 7%. Delivery of DAPT_RBC_ with fewer beams was 24% faster than plans with conventional arrangement.

**Conclusions:**

Our delivery efficiency study shows that DAPT can allow fewer beams to achieve faster delivery, as shown in case of DAPT_RBC_ workflow, and a reduction in the dose to normal tissue.

## Highlights

• Daily adaptive proton therapy may outperform offline triggered adaptation.
• Reducing beam numbers in adaptive workflows can shorten treatment time.
• Reducing margin alongside beam reduction and adaptation maintains treatment quality.

## Graphical abstract

**Figure:**
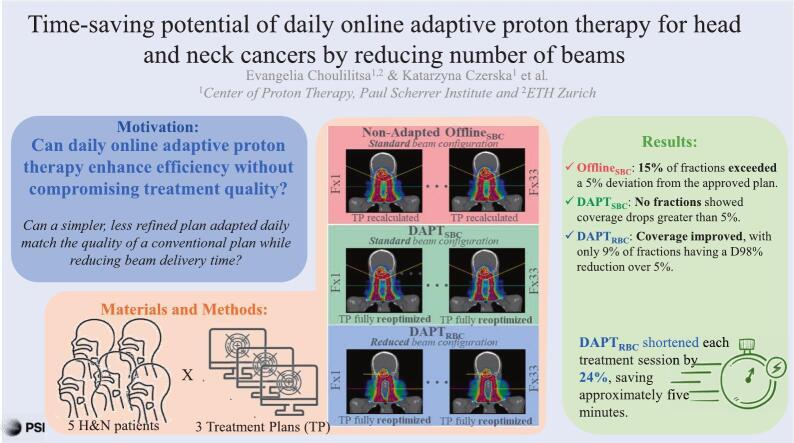

## 1 Introduction

Minor changes in the patient’s position or anatomy during delivery of radiotherapy can cause large discrepancies in planned dose distribution, particularly for particle radiotherapy [[1], [2], [3], [4]]. Adaptive proton therapy addresses these challenges by adjusting treatment plans to patient’s daily anatomy to account for these variations and ensuring consistent delivery of the prescribed dose [[5], [6], [7], [8], [9]].

Head and neck cancer (HNC) patients often experience significant anatomical changes during treatment, such as tumor shrinkage, changes in nasal cavity fillings, and weight loss [10,11]. To address these changes, the standard of care involves periodic volumetric imaging to assess their impact on dose distributions, followed by offline adaptation when necessary [12]. Daily online adaptive proton therapy (DAPT) offers a more responsive approach, updating plans based on daily imaging. Although fully online adaptive proton therapy (PT) is not yet widely available, the first clinical implementation was recently reported [13], and as technology progresses, online adaptation is expected to become the standard of care for managing anatomical uncertainties in proton therapy [12].

The clinical rationale for online adaptive therapy is twofold: it enhances treatment efficacy by ensuring target coverage despite anatomical changes, allowing for dose escalation when anatomy is favorable [6,14,15], and it reduces normal tissue toxicity by limiting exposure to OARs and minimizing normal tissue irradiation [16,17]. It may also enhance resource efficiency through hypofractionation [18] and fewer pre-treatment simulations [19]. Another cost-saving strategy is to shorten overall treatment time, including delivery, an area this study explores.

This paper explores how DAPT can enhance workflow efficiency without compromising treatment quality. We compare offline-triggered adaptation with two online workflows: using standard beam configuration and the other with a reduced beam setup for faster delivery. We hypothesize that a simpler, initially less refined plan, adapted daily, can maintain treatment quality while reducing beam delivery time. The study aimed to evaluate whether daily adaptation compensates for initial plan simplifications, optimizing the adaptive treatment timeline and improving delivery efficiency.

## 2 Materials and methods

### 2.1 Patient cohort

This retrospective analysis included five patients with HNC who were previously treated at the PSI Center for Proton Therapy (CPT). Patient data was anonymized, and general research consent was obtained. According to institutional policy and the Swiss Human Research Act (HRA, Art.2), no further ethics approval was required. Patients were selected to represent a variety of clinical scenarios, including those with major anatomical changes requiring replanning (n = 2) and those without. According to clinical protocols and standard of care, each patient underwent planning Computed Tomography (pCT) and Cone-Beam CT (CBCT) daily for positioning purposes. If offline replanning was necessary, e.g., due to anatomical changes, which was the case in two patients, an additional replanning CT (repCT) was acquired.

Daily CBCT images were acquired with an on-board imaging system on ProBeam® Proton Therapy System (Varian Medical Systems, Inc., Palo Alto, California) using image gently protocol (80kVp, 548mAs, field-of-view = 28.9 cm). Daily synthetic CTs (synCTs) were generated by deforming the pCT to match each CBCT using Velocity® (Varian Medical Systems, Inc., Palo Alto, California). These synCTs retained the Hounsfield Units (HUs) from the pCT and incorporated the anatomical details from the CBCT in overlapping regions.

The accuracy of the synCTs for each fraction was visually verified, and the original contours from the planning CT were propagated to each synCT using a B-spline deformation algorithm (Plastimatch). Additional patient details are in Table 1.

**Table 1:** Summary of each patient’s indication, number of images, offline replanning (N = number of offline-triggered adaptation), and beam/couch arrangement for each treatment scenario. Beam arrangements: anterior (A), posterior (P), anterior-oblique (AO), left-oblique (LO), right-oblique (RO), left-posterior-oblique (LPO), left-anterior-oblique (LAO), right-posterior-oblique (RPO), right-anterior-oblique (RAO).

Patient	Indications	# of daily CBCTs	Offline adaptation	Beam arrangement		
				Offline_SBC_ (PTV=CTV + 4/5mm)	DAPT_SBC_ (PTV=CTV + 2 mm)	DAPT_RBC_ (PTV=CTV + 2 mm)
P1	Squamous Cell Carcinoma Nasopharynx	27	No	P LPO LAO A RAO RPO	P LPO LAO A RAO RPO	LAO A RAO AO
P2*	Adenoid Cystic Carcinoma (ACC) Hard Palate	33	N = 2 fx9, fx21	LO LO LAO RAO RO RO	LO LO LAO RAO RO RO	LO LO RO RO
P3	Squamous Cell Carcinoma Ethmoidal	33	No	RO RAO LAO LO	RO RAO LAO LO	RO LO AO
P4*	Hypopharyngeal Squamous Cell Carcinoma, Nasal	33	N = 2 fx6, fx28	LO LO RO RO	LO LO RO RO	LO RO RO
P5	Sinonasal Undifferentiated Carcinoma (SNUC)	31	No	LO LO LAO RAO RO RO	LO LO LAO RAO RO RO	LO LAO RAO RO

### 2.2 Treatment plan

An in-house developed treatment planning system, FionA [13], was used to create clinically plausible IMPT plans using a simultaneous integrated boost (SIB) approach and following the DAHANCA guidelines for HNC [20]. For each patient, three reference treatment plans were created, two with standard beam configuration (SBC) and one with a reduced beam configuration (RBC). Beams directions were based on a clinical judgment to ensure critical structures avoidance while minimizing the beam path and integral dose to normal tissue (body−CTV1). This novel beam arrangement could better spare OARs and improve target coverage for some patients.

Three plans were prepared per patient: Offline_SBC_, a SIB plan with CTV-to-PTV = 4–5 mm and SBC (4–6 fields), DAPT_SBC_, a SIB plan with 3% range robustness, CTV-to-PTV = 2 mm, and SBC (4–6 fields), and DAPT_RBC_, a SIB plan with 3% range robustness, CTV-to-PTV = 2 mm, and RBC (3–4 fields). The Offline_SBC_ plan followed the clinical routine at our institution [21,22]. The DAPT plans were optimized on a 2 mm CTV expansion, with 3% range robustness settings, following the hybrid approach proposed by Tomassino et al. [23]. Reduced margins settings are justified given that the plan is daily reoptimized as previously reported [[24], [25], [26]].

All plans were optimized to achieve a uniform biologically weighted dose of 54.12 Gy(RBE)^2^ to CTV1, 59.40 Gy(RBE) to CTV2 and 69.96 Gy(RBE) to CTV3 in 33 fractions (RBE = 1.1) [27], except for P3 who had only two target levels (i.e., CTV1, CTV3). Target coverage in each case aimed to achieve selected clinical goals, following clinical practice at our institution: D_Mean_ ≥ 100%, D_Min_ ≥ 90%, V_95%_ ≥ 95% and D_98%_ ≥ 98% with D_Max_ < 110%. Specific OARs constraints are reported in the Supplementary Material A.

In Table 1, in addition to the patient information, we present the selected beam arrangement for Offline_SBC_, DAPT_SBC_ and DAPT_RBC_ plans with respect to patient’s anatomy.

### 2.3 Adaptive workflow

For both DAPT_SBC_ and DAPT_RBC_, all settings from the initial plan, such as beam arrangement, optimization constraints, and plan normalization, were transferred to the daily plan and reoptimized on subsequent new images (fractions: 1–33) [13,26,28]. The daily reoptimization was based on a fast analytical GPU-based raycasting dose calculation algorithm, allowing a new spot list with different pencil beam positions and fluences compared to the original template. For more details, see Matter et al. [28].

As a baseline, an offline-triggered adaptation workflow, Offline_SBC_, was simulated by recomputing the initial dose on the daily anatomy. In our clinical workflow, triggering offline adaptation is guided by dose assessments on repeat-CTs. For the simulations, offline-adapted plans were used from the specific fractions following the clinical treatment. Thus, the Offline_SBC_ plan was reoptimized on the corresponding repeat-CTs and then recalculated on each subsequent synCTs. If a patient had less than 33 synCTs, the last available synCT was used to substitute the remaining fractions.

**Fig. 1:**
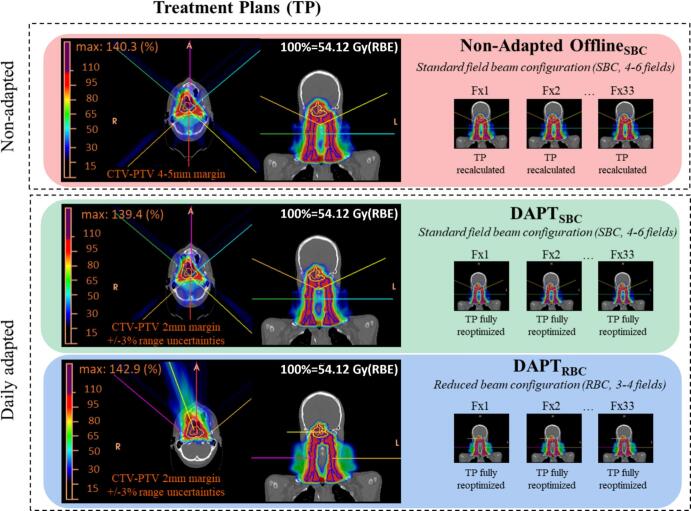
Three treatment plans were created for each patient: Offline_SBC_, with the plan being recalculated on each daily image, and two plans serving as a basis for a daily full reoptimization (DAPT_SBC_, DAPT_RBC_).

In total, two patients underwent offline replanning twice due to significant anatomical changes. Fig. 1 shows an overview of all simulated workflows.

### 2.4 Evaluation

Dose differences among all workflows were evaluated both, on an individual fraction level and for a full treatment scenario by performing dose accumulation. The dose was accumulated by inversely warping the doses calculated or reoptimized on the daily synCTs back to their corresponding pCT, using the inverted vector field used to propagate the daily structures. The DVH metrics were used to evaluate the dose differences between the workflows for all three plans.

The doses achieved for the Offline_SBC_ were used as a baseline to report fractional and cumulative results for all three workflows. Moreover, a 5% threshold was used to identify acceptable differences between daily achieved values with respect to the baseline plan [13]. This threshold helped capture fluctuations in daily doses, which are likely to be minimized when considering the cumulative dose results. Statistical significance was assessed using the Wilcoxon signed-rank test (p < 0.05) in GraphPad Prism 8.1. For daily doses, DVH curves for selected OARs were calculated for all 33 fractions per patient, showing the average ± standard deviation (SD).

Normal tissue complication probability (NTCP) for dysphagia and xerostomia (grades >2, >3) was calculated for all three treatment plans based on LIPPv2.2 models [29]. Additionally, the average dose (AD) to normal tissue was calculated as the integral dose (ID) divided by structure volume, following Salamekh et al. [30].

### 2.5 Time evaluation

For the simulated treatment scenarios, a time evaluation was performed to estimate whether the use of RBC would allow time savings during delivery, especially regarding the necessary couch movements and dose fields to be delivered. A dry run of the machine-specific files without beam delivery, including all movements and dose fields for the reference plans Offline_SBC_, DAPT_SBC,_ and DAPT_RBC_ was performed, and the time was reported.

## 3 Results

### 3.1 Target coverage

**Fig. 2:**
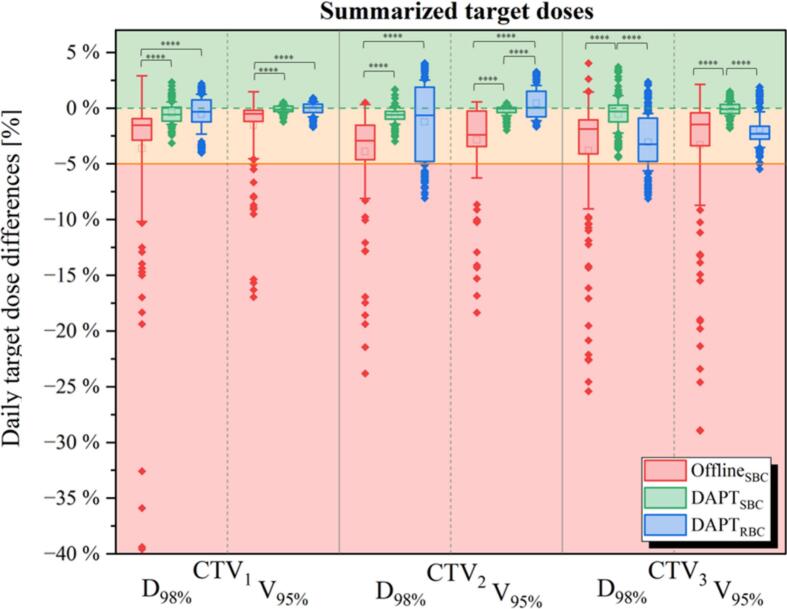
Daily target coverage differences (D98%, V95%) for three targets, summarized across all patients. Statistically significant differences are shown with four asterisks (p < 0.05). The median is represented by the horizontal lines, and the average by a small box.

Fig. 2 presents pooled data of daily target coverage differences (up to ∼40%) combined across all three CTVs and patients (each patient contributing 33 fractions, totaling 165 fractions), focusing on D98% and V95% results. In the offline-triggered adapted scenario, 15% of all fractions exceeded 5% deviation from the approved treatment plan values (9% for D_98%_ and 6% for V_95%_). In DAPT_SBC_, target coverage was never compromised and none of the target metrics exhibited a dose difference greater than 5%. Also, DAPT_RBC_ plans improved daily target coverage, with only 9% of all fractions having a reduction in the D_98%_ of more than 5% (maximum observed underdosage: 8%) and only 2 fractions with reduction in V_95%_ larger than 5%.

**Fig. 3:**
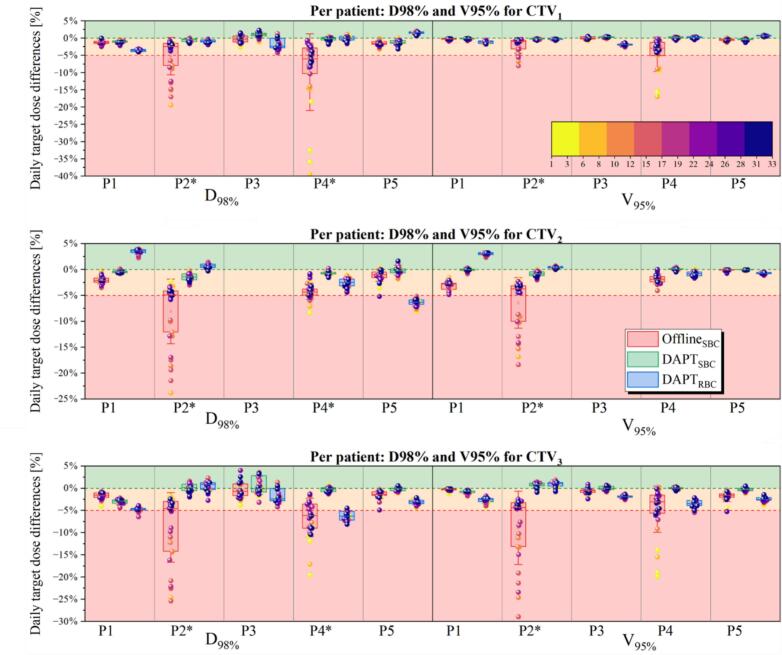
Daily differences for three targets and both, D_98%_ and V_95%_ metrics, per patient. Patients have been sorted based on target underdosage with the fraction number indicated in the color bar.

Fig. 3 presents detailed target coverage results on per-patient basis. For two patients who required offline replanning (P2, P4), both adaptive approaches restored approved target coverage. The Offline_SBC_ workflow resulted in severe underdosage in D_98%_ up to 40% and in V_95%_ of 30% for more than 33% of fractions for both patients. This underdosage mainly occurred during the period when the need for adaptation had been identified, but the new plan had not yet been implemented. The DAPT_SBC_ workflow consistently ensured satisfying daily target coverage, with differences in D_98%_ and V_95%_ never exceeding the 5% threshold. Interestingly, for these two patients, the DAPT_RBC_ approach, despite reduced and unconventional beam arrangement, outperformed the offline-triggered adapted scenario. For example, for P2, D_98%_ for all targets with the DAPT_RBC_ approach had no fractions with differences greater than 5%, whereas the Offline_SBC_ workflow resulted in 34% of fractions with differences >5% and an underdosage of up to ∼25%.

The target coverage of all the CTVs was maintained for both workflows (DAPT_SBC_, DAPT_RBC_) and patients that did not require offline replanning with the standard beam arrangement. For one patient however (P5), the initial CTV2 target coverage with DAPT_RBC_ was not as good as for the SBC and this was consistent through the fractions.

### 3.2 Daily doses to the organs-at-risk

**Fig. 4:**
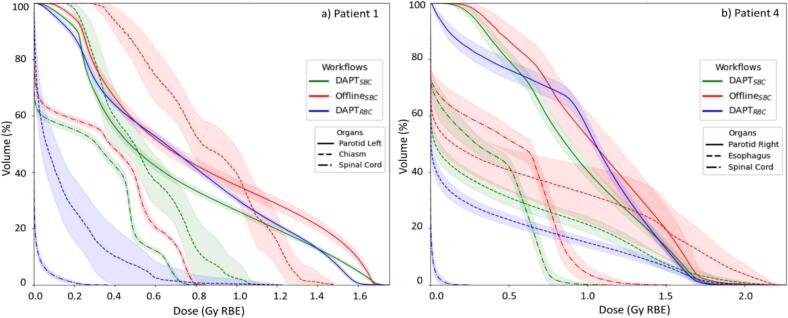
For each patient, all 33 daily DVHs are calculated for each selected organ, averaged into a mean DVH curve with SD plotted as a color-band. This process is repeated for each workflow; a) patient without offline replanning (P1), b) patient with offline replanning (P4).

Daily, the DAPT_SBC_ approach spared or provided similar doses to the baseline Offline_SBC_ plan, whereas the DAPT_RBC_ approach behaved less consistently, with some organs receiving lower doses than Offline_SBC_ and others receiving higher doses, which could be a result of reduced number of beams and different beam incidence. In Fig. 4, we plot the average DVH curve (±SD) for a selection of OARs for patient with offline replanning (P4) and patient without it (P1). In Supplementary Material B we present the results for the remaining patients.

For patients with offline replanning (P2, P4), the DAPT_SBC_ approach spared the OARs where possible. For example, in P4 (Fig. 4b), all OARs showed lower dose compared to Offline_SBC_, while target dose was restored daily. The DAPT_RBC_ delivered a higher mean dose to the left parotid gland (0.7 Gy(RBE) vs. 0.6 Gy(RBE) with Offline_SBC_), while the right parotid D_Mean_ was approximately 1.0 Gy(RBE) for both Offline_SBC_ and DAPT_SBC_. Doses to esophagus, larynx, and spinal cord were reduced daily by 0.3 Gy(RBE), 0.2 Gy(RBE), and 1.1 Gy(RBE) on average, respectively, with DAPT_SBC_ when compared to Offline_SBC_.

For patients without offline replanning (P1, P3, P5), both adaptive workflows also consistently outperformed Offline_SBC_ in OAR sparing, as shown by higher DVH curves for Offline_SBC_ across all fractions (e.g., Fig. 4a).

### 3.3 Integral Dose, NTCP and accumulated dose

Reducing the margins in the DAPT_SBC_ approach resulted in an integral dose (ID) reduction of 13% on average [range: 9%–17%] pooled across all patients, with respect to the plan used for the offline-triggered adaptive workflow. Moreover, the plan optimized using RBC resulted in an ID reduction of 15% on average across all patients [range: 0–26%] with respect to the Offline_SBC_.

Xerostomia for patients P1, P2, P4, and P5, and dysphagia for P3 were analyzed. The DAPT_SBC_ treatment scenario resulted in pooled average NTCP reductions of 7% and 9% for grade 2 and 3 toxicities, respectively, with respect to the Offline_SBC_. Similar pooled average reductions of 6% and 7% for grade 2 and grade 3 toxicities, respectively, were observed with the DAPT_RBC_ relative to Offline_SBC_. Detailed results are collected in Supplementary Material C.

Cumulative dose analysis demonstrated that both adaptive approaches achieved similar or improved target coverage compared to Offline_SBC_ (Supplementary Material D). Interestingly, in addition to satisfying coverage results, OARs sparing was significantly enhanced with the adaptive workflows with spinal cord dose reductions of up to 9.0 Gy(RBE) and 33.1 Gy(RBE) observed for P4 and DAPT_SBC_ and DAPT_RBC_, respectively.

### 3.4 Delivery time

Table 2 summarizes the time needed for each step of the delivery. Using RBC allowed us to reduce the time needed for couch movements on average by 27% [range: 13%-41%]. The dose delivery was itself reduced by ∼2 min. [range: 0.8–3 min.] when compared to the conventional beam configuration approach, and the movement by ∼3 min. [range: 1.5–5.2 min.]. In summary, with DAPT_RBC_, the overall reduction of a treatment fraction resulted in a 24% reduction, corresponding to ∼5 min.

**Table 2:** Treatment times, including couch and dose fields, are reported in minutes based on generated machine files for all scenarios. The percentage reduction for RBC is calculated for each patient by comparing the time needed for the DAPT_RBC_ vs. DAPT_SBC_.

	Time [min]											
	MOVEMENT fields				DOSE fields				TOTAL			
Patient	Offline_SBC_	DAPT_SBC_	DAPT_RBC_	Reduction by	Offline_SBC_	DAPT_SBC_	DAPT_RBC_	Reduction by	Offline_SBC_	DAPT_SBC_	DAPT_RBC_	Reduction by
1	12.9	12.9	7.6	41%	8.3	7.0	4.7	33%	21.1	19.9	12.3	38%
2	14.3	14.3	11.2	22%	6.9	6.4	5.4	16%	21.2	20.7	16.5	20%
3	11.7	11.7	10.2	13%	2.7	2.4	1.9	22%	14.3	14.1	12.1	14%
4	10.4	10.4	7.0	33%	6.3	5.9	5.1	14%	16.7	16.3	12.1	26%
5	12.7	12.7	9.5	25%	7.5	7.2	6.1	16%	20.2	19.9	15.6	22%

## 4 Discussion

This study explored simplifying beam arrangements in online daily adaptive proton therapy to enhance delivery efficiency. By reducing the number of beams and adjusting beam directions based on clinical judgment, we achieved significant reductions in OAR doses and treatment time (average = 5 min.), without compromising target coverage.

While most studies on DAPT focus primarily on dose comparisons, our analysis emphasizes the importance of workflow efficiency. Typically, online adaptive and offline-triggered adaptive treatment plans in comparative studies maintain the same beam arrangements per institutional practice [24,[31], [32], [33], [34], [35], [36], [37], [38]]. We demonstrated that modifying beam arrangements in adaptive workflows can significantly reduce treatment time. Specifically, the DAPT_RBC_ plan required 24% less gantry time than DAPT_SBC_ and outperformed offline-triggered replanning for P2 and P4 in terms of target coverage, while reducing treatment time by 20% and 26%, respectively.

Time savings are critical in online adaptive workflows, particularly for complex sites like the head and neck, where anatomical changes and critical structures require careful review [39,40]. Current workflows are not fully automated or integrated into clinical platforms, with many steps remaining manual and disjointed, limiting scalability [12,41]. While reducing beam numbers alone cannot resolve these inefficiencies, our DAPT_RBC_ approach offers a complementary strategy, enhancing clinical throughput, reducing treatment delays, and minimizing NTCP risks, especially in resource-limited proton therapy [42]. Selecting reduced beam angles through emerging automated approaches could further enhance workflow efficiency and treatment quality [43,44].

Our study followed a contour propagation workflow similar to those in the literature [24,[31], [32], [33],^45^]. While uncertainties related to deformable image registration (DIR) were not addressed, future work could investigate these uncertainties as proposed in recent studies [[46], [47], [48], [49]].

Recent studies on offline-triggered and offline-scheduled adaptation report promising outcomes, yet still underperform compared to online daily adaptive strategies [31,38,50]. Incorporating predicted anatomical changes into optimization has been shown to reduce the frequency of daily adaptations in many cases [11,[51], [52], [53]]. However, these methods rely on accurate predictions but may fail in unexpected cases [51,54].

In summary, this study demonstrates that online daily adaptive proton therapy can enable the use of reduced beam numbers, leading to shorter delivery times without compromising treatment quality. The potential for increased delivery efficiency, shown by the 24% reduction in treatment time, is promising, but further evaluation with a larger patient cohort is necessary to assess its full clinical potential.

## Funding statement

EC has received funding from the European Union’s Horizon 2020 Marie Skłodowska-Curie Actions under Grant Agreement No. 955956. KC has received funding from the European Union’s Horizon 2020 research and innovation program under the Marie Skłodowska-Curie Grant Agreement No. 884104 (PSI-FELLOW-III-3i), Grant Agreement No. 955956 (RAPTOR) and the Krebsliga grant (KFS-5660-08-2022).

## CRediT authorship contribution statement

**Evangelia Choulilitsa:** Investigation, Data curation, Methodology, Formal analysis, Validation, Visualization, Writing – original draft. **Katarzyna Czerska:** Investigation, Methodology, Formal analysis, Validation, Writing – original draft. **Barbara Bachtiary:** Validation, Writing – review & editing. **Damien Charles Weber:** Resources, Validation, Writing – review & editing. **Antony John Lomax:** Methodology, Visualization, Writing – review & editing. **Francesca Albertini:** Conceptualization, Funding acquisition, Methodology, Resources, Supervision, Writing – review & editing.

## Declaration of competing interest

The authors declare that they have no known competing financial interests or personal relationships that could have appeared to influence the work reported in this paper. Francesca Albertini is a Guest Editor for Physics and Imaging in Radiation Oncology and was not involved in the editorial review or the decision to publish this article.

## Footnotes

2 According to the International Commission on Radiation Units & Measurements (ICRU) Report 93 [27].
