# Supplementary material for: Time-saving potential of daily online adaptive proton therapy for head and neck cancers by reducing number of beams
Source: Phys Imaging Radiat Oncol. 2025 Oct 15;36:100853. doi: 10.1016/j.phro.2025.100853 (PMC12593431; doi:10.1016/j.phro.2025.100853)
Supplement: Supplementary Data 1 [file mmc1.pdf]

## Supplementary Material A

**Table A1** Prescription constraints and clinical goals for patient P1 used in the study.

| Organ at risk | Constraint |
|---|---|
| Brainstem | DMax < 60.0 Gy(RBE) |
| Brainstem Center | DMax < 54.0 Gy(RBE) |
| Chiasm | DMax < 54.0 Gy(RBE) |
| Cochlea L | DMax < 20.0 Gy(RBE) |
| Cochlea R | DMax < 20.0 Gy(RBE) |
| Esophagus | DMax < 54.0 Gy(RBE) |
| Eye L | DMax < 50.0 Gy(RBE) |
| Eye R | DMax < 50.0 Gy(RBE) |
| Lacrimal Gland L | DMax < 35.0 Gy(RBE) |
| | DMean < 25.0 Gy(RBE) |
| Lacrimal Gland R | DMax < 35.0 Gy(RBE) |
| | DMean < 25.0 Gy(RBE) |
| Larynx | DMax < 45.0 Gy(RBE) |
| Lens L | DMax < 7.0 Gy(RBE) |
| Lens R | DMax < 7.0 Gy(RBE) |
| Optic Nerve L | DMax < 54.0 Gy(RBE) |
| Optic Nerve R | DMax < 54.0 Gy(RBE) |
| Oral Cavity | DMean < 30.0 Gy(RBE) |
| Parotid L | DMax < 30.0 Gy(RBE) |
| | DMean < 25.0 Gy(RBE) |
| Parotid R | DMax < 30.0 Gy(RBE) |
| | DMean < 25.0 Gy(RBE) |
| Pharynx | DMax < 72.1 Gy(RBE) |
| Pituitary | DMean < 45.0 Gy(RBE) |
| Spinal Cord | DMax < 50.0 Gy(RBE) |
| Submandibularis L | DMax < 35.0 Gy(RBE) |
| | DMean < 30.0 Gy(RBE) |
| Submandibularis R | DMax < 35.0 Gy(RBE) |
| | DMean < 30.0 Gy(RBE) |
| Temporal Lobe L | DMax < 72.1 Gy(RBE) |
| Temporal Lobe R | DMax < 72.1 Gy(RBE) |
| Thyroid | DMax < 30.0 Gy(RBE) |
| | DMean < 15.0 Gy(RBE) |
| TMJ L | DMax < 54.0 Gy(RBE) |
| TMJ R | DMax < 54.0 Gy(RBE) |

**Table A2** Prescription constraints and clinical goals for patient P2 used in the study.

| Organ at risk | Constraint |
|---|---|
| Brainstem | DMax < 60.0 Gy(RBE) |
| Brainstem Center | DMax < 53.0 Gy(RBE) |
| Chiasm | DMax < 54.0 Gy(RBE) |
| Cochlea L | DMean < 45.0 Gy(RBE) |
| Cochlea R | DMean < 45.0 Gy(RBE) |
| Esophagus | DMean < 45.0 Gy(RBE) |
| Eye L | DMax < 45.0 Gy(RBE) |
| Eye R | DMax < 45.0 Gy(RBE) |
| Lacrimal Gland L | DMean < 25.0 Gy(RBE) |
| Lacrimal Gland R | DMean < 25.0 Gy(RBE) |
| Larynx | DMean < 40.0 Gy(RBE) |
| Lens L | DMean < 7.0 Gy(RBE) |
| Lens R | DMean < 7.0 Gy(RBE) |
| Optic Nerve L | DMax < 54.0 Gy(RBE) |
| Optic Nerve R | DMax < 60.0 Gy(RBE) |
| Oral Cavity | DMean < 30.0 Gy(RBE) |
| Parotid L | DMean < 20.0 Gy(RBE) |
| Parotid R | DMean < 20.0 Gy(RBE) |
| Pharynx | DMean < 50.0 Gy(RBE) |
| Spinal Cord | DMax < 45.0 Gy(RBE) |
| Submandibularis L | DMean < 26.0 Gy(RBE) |
| Submandibularis R | DMean < 26.0 Gy(RBE) |
| Temporal Lobe R | DMax < 72.06 Gy(RBE) |
| | D1.0cc < 74.0 Gy(RBE) |
| Thyroid | V50% < 42.0 Gy(RBE) |
| | DMean < 40.0 Gy(RBE) |
| TMJ L | DMax < 72.1 Gy(RBE) |
| TMJ R | DMax < 72.1 Gy(RBE) |

**Table A3** Prescription constraints and clinical goals for patient P3 used in the study.

| Organ at risk | Constraint |
|---|---|
| Brainstem Center | DMax < 54.0 Gy(RBE) |
| Brainstem Surface | DMax < 63.0 Gy(RBE) |
| Chiasm | DMax < 54.0 Gy(RBE) |
| Cochlea L | DMax < 40.0 Gy(RBE) |
| Cochlea R | DMax < 40.0 Gy(RBE) |
| Lacrimal Gland L | DMax < 35.0 Gy(RBE)<br>DMean < 25.0 Gy(RBE) |
| Lacrimal Gland R | DMax < 35.0 Gy(RBE)<br>DMean < 25.0 Gy(RBE) |
| Lens L | DMax < 7.0 Gy(RBE) |
| Lens R | DMax < 7.0 Gy(RBE) |
| Macula | DMax < 45.0 Gy(RBE) |
| Optic Nerve L | DMax < 58.0 Gy(RBE) |
| Optic Nerve R | DMax < 60.0 Gy(RBE) |
| Pharynx Const S | DMax < 46.0 Gy(RBE)<br>DMean < 32.0 Gy(RBE) |
| Pituitary | DMax < 35.0 Gy(RBE) |
| Temporal Lobe L | DMax < 72.1 Gy(RBE) |
| Temporal Lobe R | DMax < 72.1 Gy(RBE) |

**Table A4** Prescription constraints and clinical goals for patient P4 used in the study.

| Organ at risk | Constraint |
| --- | --- |
| Carotid L | DMax < 40.0 Gy(RBE) |
| Carotid R | DMax < 40.0 Gy(RBE) |
| Esophagus | DMean < 30.0 Gy(RBE) |
| Larynx | DMean < 40.0 Gy(RBE) |
| Oral Cavity | DMean < 30.0 Gy(RBE) |
| Parotid L | DMean < 20.0 Gy(RBE) |
| Parotid R | DMean < 20.0 Gy(RBE) |
| Pharynx Const I | DMean < 55.0 Gy(RBE) |
| Pharynx Const M | DMean < 55.0 Gy(RBE) |
| Pharynx Const S | DMean < 55.0 Gy(RBE) |
| Spinal Cord | DMax < 60.0 Gy(RBE) |
| Submandibularis L | DMean < 30.0 Gy(RBE) |
| Submandibularis R | DMean < 30.0 Gy(RBE) |
| Thyroid | DMean < 40.0 Gy(RBE) |
| TMJ L | DMax < 68.0 Gy(RBE) |
| TMJ R | DMax < 68.0 Gy(RBE) |

**Table A5** Prescription constraints and clinical goals for patient P5 used in the study.

| **Organ at risk** | **Constraint** |
|---|---|
| Brainstem Center | DMax < 54.0 Gy(RBE) |
| Brainstem Surface | DMax < 60.0 Gy(RBE) |
| Buccal Mucosa L | DMean < 30.0 Gy(RBE) |
| Buccal Mucosa R | DMean < 40.0 Gy(RBE) |
| Chiasm | DMax < 56.0 Gy(RBE) |
| Cochlea L | DMean < 36.0 Gy(RBE) |
| | D5.0% < 55.0 Gy(RBE) |
| Cochlea R | DMean < 45.0 Gy(RBE) |
| | D5.0% < 55.0 Gy(RBE) |
| Esophagus | DMean < 30.0 Gy(RBE) |
| Eye L | DMax < 50.0 Gy(RBE) |
| Eye R | DMean < 35.0 Gy(RBE) |
| Hippocampus L | D40.0% < 7.2 Gy(RBE) |
| Hippocampus R | D40.0% < 7.2 Gy(RBE) |
| Lacrimal Gland L | DMean < 25.0 Gy(RBE) |
| Lacrimal Gland R | DMean < 25.0 Gy(RBE) |
| Larynx | DMean < 40.0 Gy(RBE) |
| Lens L | DMean < 7.0 Gy(RBE) |
| Lips | DMean < 20.0 Gy(RBE) |
| Macula L | DMean < 35.0 Gy(RBE) |
| Macula R | DMean < 45.0 Gy(RBE) |
| Mandible | DMax < 72.0 Gy(RBE) |
| Optic Nerve L | DMax < 54.0 Gy(RBE) |
| Optic Nerve R | D2.0% < 72.1 Gy(RBE) |
| Oral Cavity | DMean < 30.0 Gy(RBE) |
| Parotid L | DMean < 26.0 Gy(RBE) |
| Parotid R | DMean < 26.0 Gy(RBE) |
| Pharynx Const | DMean < 55.0 Gy(RBE) |
| Retina L | DMax < 45.0 Gy(RBE) |
| Spinal Cord | DMax < 45.0 Gy(RBE) |
| Submandibularis L | DMax < 54.0 Gy(RBE) |
| Submandibularis R | DMax < 54.0 Gy(RBE) |
| Temporal Lobe L | D1.0cc < 74.0 Gy(RBE) |
| Temporal Lobe R | D1.0cc < 74.0 Gy(RBE) |
| Temporal Retina R | DMax < 54.0 Gy(RBE) |
| Thyroid | DMean < 40.0 Gy(RBE) |
| TMJ L | DMax < 68.0 Gy(RBE) |
| TMJ R | DMax < 68.0 Gy(RBE) |

## Supplementary Material B

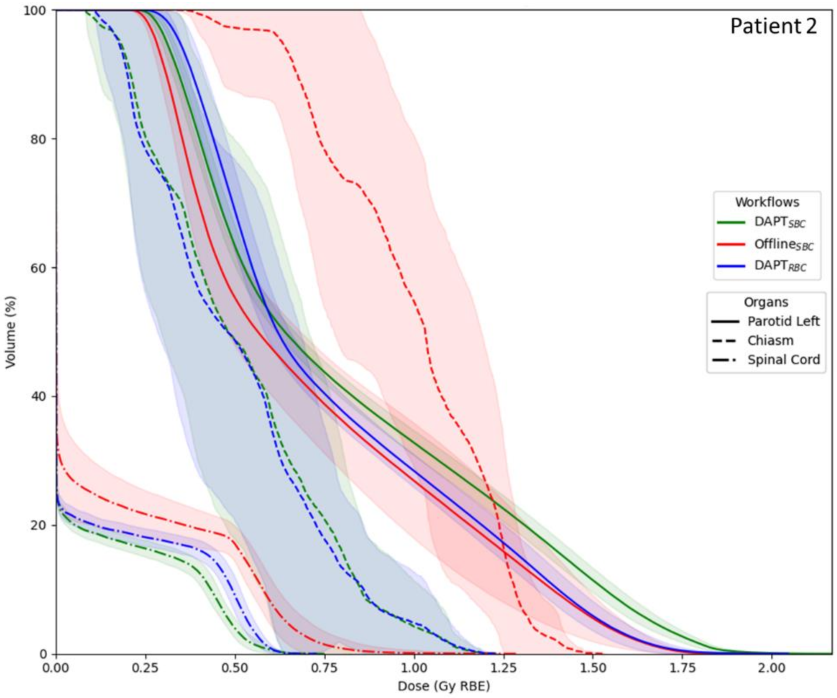


**Figure B1** Average DVH curve (±SD) for selected OARs for patient P2 (with offline replanning).

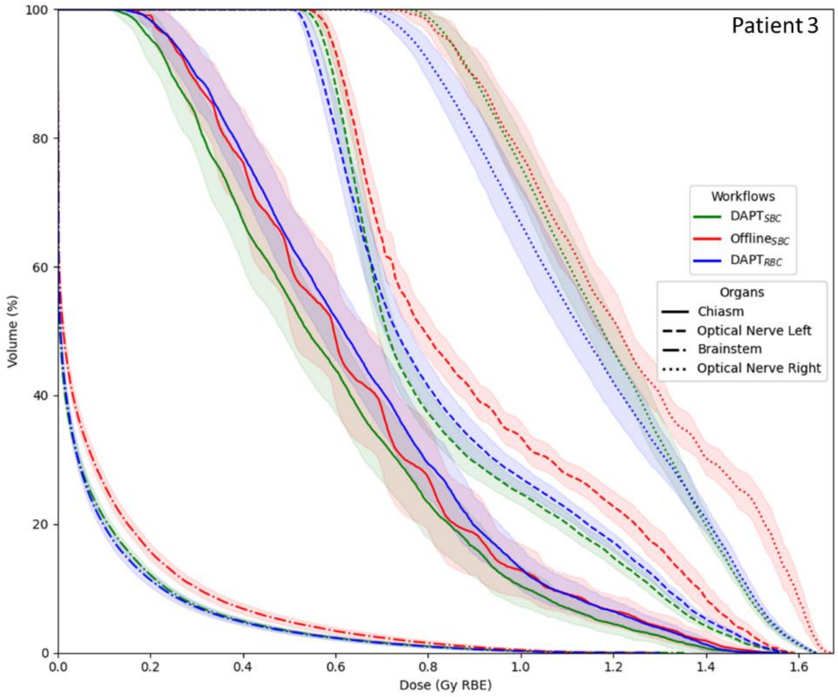


**Figure B2** Average DVH curve (±SD) for selected OARs for patient P3 (without offline replanning).

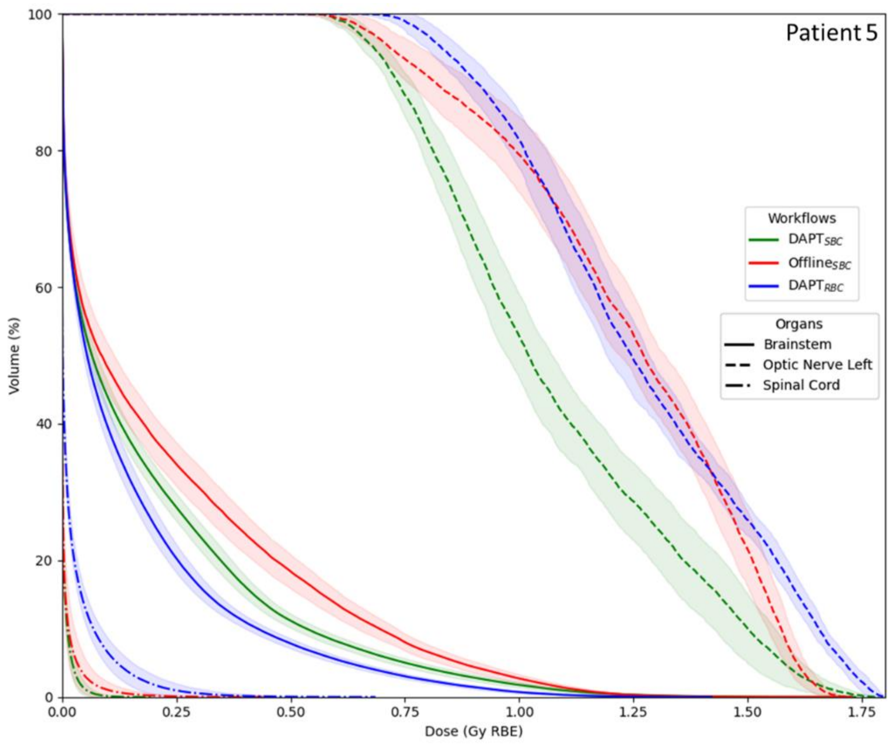


**Figure B3** Average DVH curve (±SD) for selected OARs for patient P5 (without offline replanning).

## Supplementary Material C

**Table C1** Average dose reduction across all three planning approaches on a per-patient basis. The average, minimum, and maximum average dose reductions are also shown.

| | **Integral dose reduction** | | |
|---|---|---|---|
| | *$DAPT_{SBC}$ vs $Offline_{SBC}$* | *$DAPT_{RBC}$ vs $Offline_{SBC}$* | *$DAPT_{RBC}$ vs $DAPT_{SBC}$* |
| **patient 1** | 16.7% | 25.0% | 10.0% |
| **patient 2** | 16.7% | 16.7% | 0.0% |
| **patient 3** | 11.1% | 0.0% | -12.5% |
| **patient 4** | 10.5% | 26.3% | 17.6% |
| **patient 5** | 9.1% | 4.5% | -5.0% |
| **average** | 12.8% | 14.5% | 2.0% |
| **minimum** | 9.1% | 0.0% | -12.5% |
| **maximum** | 16.7% | 26.3% | 17.6% |

**Table C2** Reduction in NTCP values on a per patient basis for each of the approaches used in this study. For each NTCP (both Grade 2 and Grade 3), we calculate the reduction per patient. We also calculate the average, minimum, and maximum values.

| | **Patient relevant NTCP Reduction for $DAPT_{SBC}$ vs $Offline_{SBC}$** | | **Patient relevant NTCP Reduction for $DAPT_{RBC}$ vs $Offline_{SBC}$** | | **Patient relevant NTCP Reduction for $DAPT_{RBC}$ vs $DAPT_{SBC}$** | |
|---|---|---|---|---|---|---|
| | *Grade 2* | *Grade 3* | *Grade 2* | *Grade 3* | *Grade 2* | *Grade 3* |
| **patient 1** | 10.7% | 11.3% | 3.5% | 3.8% | -8.0% | -8.5% |
| **patient 2** | 3.9% | 4.5% | 4.2% | 4.8% | 0.3% | 0.4% |
| **patient 3** | 6.0% | 11.5% | 7.5% | 12.8% | 1.6% | 1.5% |
| **patient 4** | 15.2% | 16.5% | 13.3% | 14.6% | -2.2% | -2.3% |
| **patient 5** | -0.1% | -0.1% | 2.0% | 2.1% | 2.0% | 2.2% |
| **average** | 7.1% | 8.7% | 6.1% | 7.6% | -1.2% | -1.4% |
| **minimum** | -0.1% | -0.1% | 2.0% | 2.1% | -8.0% | -8.5% |
| **maximum** | 15.2% | 16.5% | 13.3% | 14.6% | 2.0% | 2.2% |

## Supplementary Material D

**Table D1** Cumulative dose results for patient P1 and all three simulated workflows, i.e., $Offline_{SBC}$, $DAPT_{SBC}$ and $DAPT_{RBC}$. Dose for targets and selected OARs reported.

| | | Cumulative dose results | | |
|---|---|---|---|---|
| | Prescription | $Offline_{SBC}$ | $DAPT_{SBC}$ | $DAPT_{RBC}$ |
| **CTV1** | D98%>98% | 98.4% | 97.9% | 95.6% |
| | V95%>95% | 99.4% | 99.3% | 98.8% |
| **CTV2** | D98%>98% | 91.4% | 92.9% | 96.1% |
| | V95%>95% | 93.6% | 96.2% | 98.6% |
| **CTV3** | D98%>98% | 96.7% | 95.0% | 94.0% |
| | V95%>95% | 98.5% | 98.1% | 96.6% |
| **Chiasm** | DMax<54.0 Gy(RBE) | 48.1 Gy(RBE) | 36.3 Gy(RBE) | 27.5 Gy(RBE) |
| **Parotid Left** | DMax<30.0 Gy(RBE) | 55.7 Gy(RBE) | 56.8 Gy(RBE) | 55.2 Gy(RBE) |
| | DMean<25.0 Gy(RBE) | 26.4 Gy(RBE) | 22.3 Gy(RBE) | 24.6 Gy(RBE) |
| **Spinal Cord** | DMax<50.0 Gy(RBE) | 26.5 Gy(RBE) | 25.4 Gy(RBE) | 7.3 Gy(RBE) |

**Table D2** Cumulative dose results for patient P2 and all three simulated workflows, i.e., $Offline_{SBC}$, $DAPT_{SBC}$ and $DAPT_{RBC}$. Dose for targets and selected OARs reported.

| | | Cumulative dose results | | |
|---|---|---|---|---|
| | Prescription | $Offline_{SBC}$ | $DAPT_{SBC}$ | $DAPT_{RBC}$ |
| **CTV1** | D98%>98% | 98.1% | 99.7% | 99.3% |
| | V95%>95% | 99.3% | 99.8% | 99.7% |
| **CTV2** | D98%>98% | 91.6% | 94.3% | 96.5% |
| | V95%>95% | 94.5% | 97.5% | 98.7% |
| **CTV3** | D98%>98% | 88.1% | 92.8% | 93.1% |
| | V95%>95% | 88.9% | 96.3% | 96.7% |
| **Chiasm** | DMax<54.0 Gy(RBE) | 43.9 Gy(RBE) | 28.2 Gy(RBE) | 28.5 Gy(RBE) |
| **Parotid Left** | DMean<20.0 Gy(RBE) | 23.7 Gy(RBE) | 26.6 Gy(RBE) | 25.4 Gy(RBE) |
| **Spinal Cord** | DMax<45.0 Gy(RBE) | 25.9 Gy(RBE) | 19.2 Gy(RBE) | 21.4 Gy(RBE) |

**Table D3** Cumulative dose results for patient P3 and all three simulated workflows, i.e., $Offline_{SBC}$, $DAPT_{SBC}$ and $DAPT_{RBC}$. Dose for targets and selected OARs reported.

| | | Cumulative dose results | | |
|---|---|---|---|---|
| | Prescription | $Offline_{SBC}$ | $DAPT_{SBC}$ | $DAPT_{RBC}$ |
| **CTV1** | D98%>98% | 98.7% | 99.4% | 98.9% |
| | V95%>95% | 99.4% | 99.5% | 99.3% |
| **CTV2** | D98%>98% | - | - | - |
| | V95%>95% | - | - | - |
| **CTV3** | D98%>98% | 92.3% | 91.5% | 89.5% |
| | V95%>95% | 96.9% | 96.8% | 95.2% |
| **Brainstem** | DMax<63.0 Gy(RBE) | 41.0 Gy(RBE) | 44.2 Gy(RBE) | 40.8 Gy(RBE) |
| **Chiasm** | DMax<54.0 Gy(RBE) | 49.0 Gy(RBE) | 48.3 Gy(RBE) | 49.9 Gy(RBE) |
| **Optical Nerve Left** | DMax<58.0 Gy(RBE) | 53.9 Gy(RBE) | 54.6 Gy(RBE) | 54.8 Gy(RBE) |
| **Optical Nerve Right** | DMax<60.0 Gy(RBE) | 56.3 Gy(RBE) | 56.2 Gy(RBE) | 55.7 Gy(RBE) |

**Table D4** Cumulative dose results for patient P4 and all three simulated workflows, i.e., $Offline_{SBC}$, $DAPT_{SBC}$ and $DAPT_{RBC}$. Dose for targets and selected OARs reported.

| | | Cumulative dose results | | |
|---|---|---|---|---|
| | Prescription | $Offline_{SBC}$ | $DAPT_{SBC}$ | $DAPT_{RBC}$ |
| **CTV1** | D98%>98% | 95.4% | 98.5% | 98.5% |
| | V95%>95% | 98.3% | 99.6% | 99.6% |
| **CTV2** | D98%>98% | 98.5% | 98.8% | 97.3% |
| | V95%>95% | 99.7% | 99.8% | 99.0% |
| **CTV3** | D98%>98% | 95.6% | 97.9% | 93.1% |
| | V95%>95% | 98.4% | 99.4% | 96.9% |
| **Esophagus** | DMean<30.0 Gy(RBE) | 15.7 Gy(RBE) | 10.8 Gy(RBE) | 8.0 Gy(RBE) |
| **Parotid Right** | DMean<20.0 Gy(RBE) | 34.3 Gy(RBE) | 30.0 Gy(RBE) | 31.5 Gy(RBE) |
| **Spinal Cord** | DMax<60.0 Gy(RBE) | 40.5 Gy(RBE) | 31.5 Gy(RBE) | 7.4 Gy(RBE) |

**Table D5** Cumulative dose results for patient P5 and all three simulated workflows, i.e., $Offline_{SBC}$, $DAPT_{SBC}$ and $DAPT_{RBC}$. Dose for targets and selected OARs reported.

| | | Cumulative dose results | | |
|---|---|---|---|---|
| | Prescription | $Offline_{SBC}$ | $DAPT_{SBC}$ | $DAPT_{RBC}$ |
| **CTV1** | D98%>98% | 96.1% | 96.4% | 99.2% |
| | V95%>95% | 98.5% | 98.6% | 99.7% |
| **CTV2** | D98%>98% | 104.8% | 105.5% | 99.1% |
| | V95%>95% | 100.0% | 99.9% | 99.4% |
| **CTV3** | D98%>98% | 93.7% | 94.4% | 91.7% |
| | V95%>95% | 96.1% | 97.3% | 94.8% |
| **Brainstem** | DMax<60.0 Gy(RBE) | 48.4 Gy(RBE) | 52.2 Gy(RBE) | 46.9 Gy(RBE) |
| **Optical Nerve Left** | DMax<54.0 Gy(RBE) | 55.3 Gy(RBE) | 54.1 Gy(RBE) | 55.8 Gy(RBE) |
| **Optical Nerve Right** | D2.0%<72.1 Gy(RBE) | 71.0 Gy(RBE) | 70.3 Gy(RBE) | 75.5 Gy(RBE) |
| **Spinal Cord** | DMax<45.0 Gy(RBE) | 8.0 Gy(RBE) | 4.1 Gy(RBE) | 15.4 Gy(RBE) |
